# Atypical preeclampsia before 20 weeks of gestation with multicystic placenta, hyperreactio luteinalis, and elevated sFlt-1/PlGF ratio as manifestations of fetal triploidy: A case report

**DOI:** 10.1016/j.crwh.2021.e00379

**Published:** 2021-12-27

**Authors:** Harue Hayashida, Koji Nakamura, Koto Ukon, Kazuaki Sato, Kazuya Mimura, Mamoru Kakuda, Aska Toda, Tatsuya Miyake, Kosuke Hiramatsu, Toshihiro Kimura, Masayuki Endo, Tadashi Kimura

**Affiliations:** a Department of Obstetrics and Gynecology, Osaka University Graduate School of Medicine, Suita, Osaka, Japan; b Department of Pathology, Osaka University Graduate School of Medicine, Suita, Osaka, Japan

**Keywords:** Preeclampsia before 20 weeks, Molar placenta, Triploidy, Hyperreactio luteinalis, sFlt-1/PlGF ratio

## Abstract

Preeclampsia is one of the most common as well as most severe complications of pregnancy, characterized by new-onset hypertension and proteinuria or other organ dysfunction. It predominantly occurs after 20 weeks of gestation. Very rarely, it can be triggered earlier in some specific situations. Here we report a case of fetal triploidy presenting as an extraordinarily early-onset preeclampsia. A healthy 36-year-old multiparous woman who had conceived naturally was hospitalized due to acute-onset severe hypertension accompanied by proteinuria at 18 weeks of gestation. Laboratory testing ruled out the presence of underlying maternal disease. Ultrasound findings, including multicystic large placenta and multiple fetal anomalies, strongly suggested fetal triploidy. Maternal ovaries showed hyperreactio luteinalis. The soluble fms-like tyrosine kinase-1/ placental growth factor (sFlt-1/PlGF) ratio was elevated, at 270. Medical abortion was carried out at 19 weeks of gestation; thereafter, her symptoms quickly resolved. Fetal triploidy was confirmed by genetic testing. We should be aware that fetal disorders including triploidy as well as pre-existing maternal diseases can provoke such very early-onset preeclampsia. Fetal ultrasound evaluation is critical and the sFlt-1/PlGF ratio is important for prompt diagnosis and management to prevent adverse maternal outcomes associated with atypical preeclampsia before 20 weeks of gestation.

## Highlights

• Fetal ultrasound and the sFlt-1/PlGF ratio are important to diagnose preeclampsia even before 20 weeks of gestation.

## 1 Introduction

Preeclampsia is one of the most common as well as critical complications during pregnancy. It is characterized by new-onset hypertension accompanied by proteinuria or other organ dysfunction after 20 weeks of gestation [[1], [2], [3], [4]]. Although preeclampsia can occur before 20 weeks [5,6], this is so rare that timely and accurate diagnosis remains challenging.

Triploidy results from an extra haploid set of chromosomes of paternal or maternal origin [7]. As most fetuses are miscarried in the first trimester, triploidy occurs in only about 0.002% of viable pregnancies between 16 and 20 weeks of gestation [8]. Triploidy of paternal origin (diandric triploidy) is reported to be associated with placental overgrowth and preeclampsia as early as the second trimester [7,9]. Here we present the case of a healthy woman who had conceived naturally and developed preeclampsia at 18 weeks of gestation as a manifestation of diandric triploidy.

## 2 Case Presentation

A 36-year-old woman (gravida 2, para 1) conceived naturally. Her medical history was unremarkable. On her initial visit at 12 weeks of gestation, her blood pressure was 103/65 mmHg, and her urinalysis was normal. At the first-trimester ultrasound screening, the thickness of the fetal nuchal translucency was 3.1 mm. She did not request further evaluation although the estimated risk of trisomy 21 was as high as 1 in 31. At her next regular checkup, at 16 weeks of gestation, her blood pressure was slightly elevated at 123/76 mmHg. Self-monitoring of blood pressure and low-dose aspirin were initiated in consideration of the risk of preeclampsia. Her blood pressure continued to increase and reached approximately 160/110 mmHg at the end of 17 weeks of gestation; however, she did not contact us. She had an unplanned visit to the hospital with complaints of headache, nausea, and severe hypertension, as high as 198/110 mmHg, at 18 weeks and 4 days of gestation. On admission, her blood pressure was 172/116 mmHg. Laboratory data were as follows: serum creatinine 46 uM/L, platelet count 178,000/μL, aspartate aminotransferase (AST) 33 U/L, alanine aminotransferase (ALT) 27 U/L, albumin 21 g/L. Her dipstick urinalysis was 1+ proteinuria.

Intravenous nifedipine and magnesium sulfate were immediately initiated to control blood pressure and to prevent eclampsia, respectively. Laboratory tests and imaging studies of her brain showed no evidence of maternal diseases which could induce hypertension or of an intracranial lesion. On the third day, the criteria for preeclampsia were met based on the 2.5 g/day of proteinuria.

**Fig. 1:**
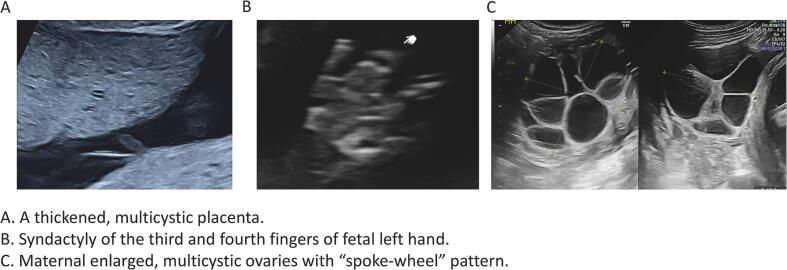
Ultrasound screening at 19 weeks of gestation.

On the fifth day, fetal ultrasound screening was performed. A thickened, multicystic placenta (Fig. 1a) and multiple fetal anomalies were detected, including cleft lip and palate, short nasal bone, pericardial effusion, and syndactyly of the third and fourth fingers (Fig. 1b). Fetal growth and amniotic fluid volume were normal. The maternal ovaries were symmetrically enlarged to 7 cm in diameter with a “spoke-wheel” pattern (Fig. 1c). The serum human chorionic gonadotropin (hCG) value was 512,652 IU/L. These findings strongly suggested diandric triploidy.

**Fig. 2:**
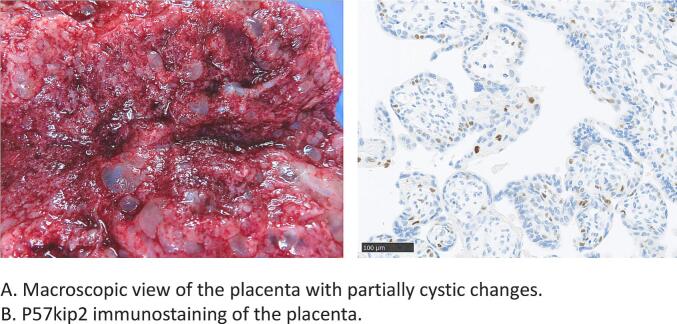
Placental pathology.

Soluble fms-like tyrosine kinase-1 (sFlt-1) and placental growth factor (PlGF) were 12,100 and 44.7 pg/mL, respectively, and the sFlt-1/PlGF ratio was 270; however, these results were obtained after delivery. Amniocentesis was considered for rapid genetic testing; however, medical termination of the pregnancy was decided upon since her dyspnea deteriorated due to pulmonary edema and her liver enzymes were elevated: AST 92 U/L and ALT 63 U/L. On the sixth day, a 224 g baby and a notably large, 400 g placenta with partial cystic changes (Fig. 2a) were vaginally delivered at 19 weeks and 2 days of gestation. Placental immunostaining showed P57Kip2 positive cells (Fig. 2b), suggesting the presence of the maternal allele. Triploidy (69, XXX) was confirmed by genetic testing of skin tissue of the affected baby. The patient's hypertension and proteinuria returned to normal at 2 weeks after delivery. The values of her serum hCG quickly declined. Although her ovaries continued to enlarge to 12 cm in diameter at 5 weeks after delivery, they had spontaneously shrunk to normal size at 16 weeks after delivery.

## 3 Discussion

Three important clinical implications of this case should be discussed. First, preeclampsia can be triggered even before 20 weeks of gestation by fetal-placental disorders as well as pre-existing maternal diseases. Second, ultrasound evaluation of the fetus is critical for prompt diagnosis and management of this condition. Third, the rapid result of the sFlt-1/PlGF ratio is important for diagnosing preeclampsia and for differentiating it from other underlying diseases.

Preeclampsia typically occurs after 20 weeks of gestation. Hypertension that occurs before pregnancy or before 20 weeks of gestation is classified as a distinct entity: chronic hypertension. However, preeclampsia can be provoked even before 20 weeks of gestation in some specific situations [5,6]. First, pre-existing diseases such as chronic kidney diseases [10], antiphospholipid syndrome [11], systemic lupus erythematosus [6] and Cushing syndrome [12] have been reported to induce preeclampsia during the first half of pregnancy. Since these complications might be newly identified during pregnancy, such women should be screened for the presence of these diseases even if they have no previous medical history. Second, fetal-placental disorders can also induce such conditions. Molar pregnancies including triploidy [9] and hydatidiform mole with or without a coexisting fetus [13,14] and trisomy 13 [15] have been reported to induce very early-onset preeclampsia. Preeclampsia induced by fetal hydrops is known as mirror syndrome [16]. Although these fetal-placental disorders are rare, they should be included as differential diagnoses when patients show features of very early-onset preeclampsia.

Ultrasound evaluation is critical for prompt diagnosis and management of such extraordinarily early-onset preeclampsia. The presence of most pre-existing maternal diseases is usually diagnosed on the basis of laboratory findings and further evaluation, occasionally in consultation with experts. Fetal-placental disorders, however, might be recognized solely on the basis of fetal ultrasound unless rapid genetic testing is performed. The most important ultrasound finding related to diandric triploidy is multicystic large placenta with a normal-sized fetus with structural anomalies [7]. Fetal structural anomalies detected in triploidy are extremely heterogenous; central nervous system anomalies are common; cardiac defects are usually severe and complex; and renal abnormalities, including renal agenesis, multicystic kidneys, and hydronephrosis, are also sometimes observed. Other minor findings, such as absence of the gall bladder, hypoplastic lungs, omphalocele, syndactyly of the third and fourth fingers or toes, club hands or feet, or polydactyly may be observed. Theca lutein cysts of both maternal ovaries, also known as hyperreactio luteinalis, are frequently seen [7]. There are other differential diagnoses in early-onset preeclampsia with multiple fetal malformations, multicystic placenta, and/or hyperreactio luteinalis. In hydatidiform mole with a coexisting fetus, a multicystic large placenta is observed though the fetus is generally normal. In trisomy 13, multiple, severe structural malformations are observed, including holoprosencephaly or other central nervous system anomalies, midline facial anomalies, and renal and cardiac defects [17]. Growth restriction is also present in about half the cases [18]. Mirror syndrome can be recognized when a hydropic fetus and placenta are observed [16]. These fetal-placental disorders are usually detectable using ultrasound. It is essential to take fetal-placental disorders into consideration as causes of very early-onset preeclampsia for timely and accurate diagnosis.

Recently, the sFlt-1/PlGF ratio has been considered as a reliable tool for diagnosing preeclampsia and differentiating it from other diseases. SFlt-1, an antagonist of PlGF, causes maternal endothelial dysfunction, resulting in the clinical findings of preeclampsia [19]. A prospective multicenter study has showed that a sFlt-1/PlGF ratio cutoff of ≤38 can rule out preeclampsia within 1 week with a negative predictive value of 99.3% [20]. The sFlt-1/PlGF ratio has been reported to be elevated even with very early-onset preeclampsia [6]. In molar pregnancy [21] and trisomy 13 [17], it has been shown to be elevated before the onset of preeclampsia. To our knowledge, this is the first case with elevated sFlt-1/PlGF ratio in diandric triploidy. The sFlt-1/PlGF ratio is important for early awareness and diagnosis of such very early-onset preeclampsia and for differentiating it from other underlying diseases. It is desirable to establish a testing system that can show the results of the sFlt-1/PlGF ratio promptly.

Although the pathogenesis and management of preeclampsia before 20 weeks of gestation is not well described even in major clinical guidelines for hypertensive disorders in pregnancy [[1], [2], [3], [4]], fetal ultrasound and the sFlt-1/PlGF ratio will provide critical information for dealing with this condition.

## 4 Conclusion

Preeclampsia can occur even before 20 weeks of gestation via fetal-placental disorders as well as pre-existing maternal diseases. Fetal ultrasound screening is critical and the sFlt-1/PlGF ratio is important for differential diagnosis of the pathogenesis and management of this condition.
